# Dissociation Constant of 4-Aminopyridinium Ion in Water From 0 to 50 °C and Related Thermodynamic Quantities

**DOI:** 10.6028/jres.064A.043

**Published:** 1960-10-01

**Authors:** Roger G. Bates, Hannah B. Hetzer

## Abstract

The dissociation constant of 4-aminopyridinium ion in water at 11 temperatures from 0° to 50° C has been determined from electromotive force measurements of 19 approximately equimolal aqueous buffer solutions of 4-aminopyridine and 4-aminopyridinium chloride. Cells without liquid junction were used; the cell is represented as follows:

$$\text{Pt};\;H_{2}(g),\;H_{2}{\text{NC}}_{5}H_{4}N\cdot \text{HCl}(m_{1}),\;H_{2}{\text{NC}}_{5}H_{4}N(m_{2}),\;\text{AgCl};\;\text{Ag}$$

where *m* is molality.

Between 0° and 50° C, the dissociation constant (*K_bh_*) is given as a function of temperature (*T*) in degrees Kelvin by

$$-\log\;K_{bh}=\frac{2575.8}{T}+0.08277+0.0013093T$$

The changes of Gibbs free energy (Δ*G*°), of enthalpy (Δ*H*°), of entropy (Δ*S*°), and of heat capacity (Δ*C_p_*°) for the dissociation process in the standard state were calculated from the constants of this equation. At 25° C the following values were found:

$$\begin{array}{l} -\log\;K_{bh}=9.114,\;\Delta G{}^{\circ}=52,013\;j\;{\text{mole}}^{-1},\;\Delta H{}^{\circ}=47,090\;j\;{\text{mole}}^{-1},\\ \;\Delta S{}^{\circ}=-16.5\;j\;\deg^{-1}\;{\text{mole}}^{-1},\;\Delta C_{p}^{{}^{\circ}}=-15\;j\;\deg^{-1}\;{\text{mole}}^{-1}. \end{array}$$

Thermodynamic constants for the basic dissociation of 4-aminopyridine at 25° C were also computed.

## 1. Introduction

The usefulness of 4-aminopyridine as a standard for the direct titration of acid solutions has been pointed out by Van Hall and Stone [1].^1^ The base is a solid substance at room temperature, melting at 161° C. It is a monoprotic base with an equivalent weight of 94.119 and can be purified easily by recrystallization from toluene or benzene or by sublimation.

Conductivity data [2, 3] and potentiometric titrations [1, 4] indicate that the negative logarithm of the basic dissociation constant is approximately 4.8 to 4.9 at 20° to 25° C. 4-Aminopyridine is accordingly about as strong as ammonia and is consequently a strong enough base to yield a sharp inflection point when titrated with a solution of a strong acid. Furthermore, the equivalence point of the titration matches the *p*H of the color transformation of methyl red sufficiently closely that this convenient indicator can be effectively utilized [1]. The base is now available commercially in pure form. One company supplies material specifically intended for use as a reference standard in acidimetry.

It appears likely, therefore, that 4-aminopyridine will find increasing use in the research laboratory. An accurate knowledge of the acidic dissociation constant of 4-aminopyridinium ion over a range of temperatures will permit this acid-base system to be used for *p*H control in the region pH 8.5 to 9.5 where the undesirable side reactions of borate buffers often preclude the use of borax or boric acid. The mechanism of the acidic dissociation of the cation acids conjugate to weak bases is a subject of continuing interest in this laboratory (see for example, ref. [5,6,7]). The changes of entropy and heat capacity calculated from the temperature coefficient of the dissociation constant offer a possible means of assessing the relative importance of the several structural factors on which the extent of acidic or basic dissociation is known to depend [8].

## 2. Determination of the Dissociation Constant

The emf method, which has been described in some detail in earlier publications [5,9,10], makes use of a cell without liquid junction:

$$\begin{array}{c} \text{Pt};H_{2}(g,1\;\text{atm}),\;H_{2}{\text{NC}}_{5}H_{4}N\cdot \text{HCl}(m_{1}),\\ H_{2}{\text{NC}}_{5}H_{4}N(m_{2}),\;\text{AgCl};\text{Ag}, \end{array}$$

where *m*_1_ and *m*_2_ represent respectively the molalities of 4-aminopyridinium chloride (BHCl) and the corresponding free base (B). The dissociation of the 4-aminopyridinium ion can be formulated

$${\text{BH}}^{+}+H_{2}O=B+H_{3}O^{+}.$$ (1)

The thermodynamic dissociation constant is represented by the symbol *K_bh_*.

Inasmuch as 4-aminopyridine is a stronger base than water, the point of equilibrium in reaction (1) lies rather far to the left. Indeed, the free base reacts with water (dissociates) to a sufficient extent that a small correction must be applied to *m*_1_ and *m*_2_ to obtain the true concentrations of BH^+^ and B. The dissociation reaction is

$$B+H_{2}O={\text{BH}}^{+}+{\text{OH}}^{-},$$ (2)

and the corrected concentrations of 4-aminopyridinium ion and 4-aminopyridine are given by

$$m_{\text{BH}+}=m_{1}+m_{\text{OH}}$$ (3)

and

$$m_{B}=m_{2}-m_{\text{OH}}.$$ (4)

In order to relate *K_bh_* in a useful way to the emf (*E*) of the cell, the mass-law expression for *K_bh_* was combined with the Nernst equation for the cell. The resulting equations are as follows:

$$\begin{array}{l} -\log\;K_{bh}-\beta m_{1}\\ =\frac{E-E{}^{\circ}}{2.3026RT/F}+\log\frac{m_{1}(m_{1}+m_{\text{OH}})}{m_{2}-m_{\text{OH}}}-\frac{2A\sqrt{m_{1}}}{1+Ba*\sqrt{m_{1}}} \end{array}$$ (5a)

$$=pwH+\log\frac{m_{1}+m_{\text{OH}}}{m_{2}-m_{\text{OH}}}-\frac{2A\sqrt{m_{1}}}{1+Ba*\sqrt{m_{1}}}.$$ (5b)

In deriving these equations, the concentrations of BH^+^ and B were expressed by eq (3) and (4), the term $f_{\text{BH}}{\;}^{+}f_{\text{Cl}}{\;}^{-}a_{H_{2}O}/f_{B}$ (where *f* is an activity coefficient on the molal scale and $a_{H_{2}O}$ is the water activity) was expressed by an equation of the Hückel form containing two parameters, α* and *β*, and the Debye- Hückel constants *A* and *B* [11], and the ionic strength was set equal to the molality of 4-aminopyridinium chloride.

For convenience, (−log *K_bh_−βm*_1_) is called $-\log\;K_{bh}^{\prime }$. It is evident that $K_{bh}^{\prime }$ is an “apparent” or inexact value of the dissociation constant which, however, becomes equal to the true value of the thermodynamic constant *K_bh_* when *m*_1_ becomes 0, that is, at infinite dilution.

In eq (5a), *E*° is the standard potential of the hydrogen-silver chloride cell [12] and *R, T*, and *F* are, respectively, the gas constant per mole, the temperature in degrees Kelvin, and the faraday. The term *pw*H., a useful experimental quantity numerically equal to −log(*f*_H_*f*_Cl_*m*_H_), was defined in an earlier publication [13]. Its equivalent in terms of the emf and chloride ion molality is readily apparent from a comparison of eq (5a) and (5b).

It has been shown [14] that the hydroxyl ion concentration can be obtained with the accuracy needed to correct the buffer ratio by

$$\log\;m_{\text{OH}}\approx \log\;K_{w}+pwH,$$ (6)

where *K_w_* is the ion product constant for water [9]. This approximation is quite satisfactory if the base is not too strong (i.e., when *m*_OH_ is not too large). It has been found to be adequate for the calculation of the dissociation constant of piperidinium ion (*m*_OH_ ≈ 0.002) [7] but fails when applied to the treatment of the data for CaOH^+^ (*m*_OH_ ≈ 0.02) [15].

## 3. Experimental Procedures

Commercial, unpurified 4-aminopyridine was recrystallized from a mixture of benzene and alcohol.^2^ It was then crystallized twice from water, crushed, and dried for four hours at 105° C as recommended by Van Hall and Stone [1]. A further drying for two hours at 110° C brought about some sublimation. The melting point was found to be 160° C at a heating rate of about 1° C min^−1^.

Hydrochloric acid of reagent grade was diluted to about 6 *M* and distilled twice, the middle third being collected in each instance. The redistilled acid was diluted to make a stock solution with a molality about 0.1 which was standardized by a gravimetric determination of chloride as silver chloride. Two different stock solutions were used in the course of the work. On the average, replicate determinations of the molality agreed within ±0.02 percent.

The assay of the purified 4-aminopyridine was determined by titration with the standard solution of hydrochloric acid. The concentration of pyridinium salt at the endpoint was about 0.035 *M*, and the *p*H was adjusted to the calculated equivalence point (*p*H 5.3) with the aid of a glass- electrode *p*H meter. The material assayed 100.0±0.1 percent.

Four stock solutions composed of 4-aminopyridine and hydrochloric acid were prepared. Each of these was diluted with distilled water to make other cell solutions, but no more than five cell solutions were prepared from each stock solution. Dissolved air was removed from the solutions by passing purified hydrogen through them for about 2 hours before the cells were filled. The cell vessels were flushed with hydrogen; the solution was then admitted and the vessel filled and emptied twice before the final filling. The preparation of the electrodes has been described elsewhere [16].

The solubility of silver chloride in a 0.1-*M* solution of 4-aminopyridine was found to be 0.00104 mole l^−1^, from which the equilibrium constant (*K_f_*) for the formation of the complex ion AgB_2_^+^ is found to be 7×10^5^. The 4-aminopyridine complex of silver ion is thus considerably less stable than the corresponding silver-ammonia complex, Ag(NH_3_)^+^_2_, for which the formation constant (*K_f_*) is about 1.6×10^7^ [17]. Bates and Pinching [5] showed that the percentage increase in *m*_Cl_ due to solubility of silver chloride is nearly proportional to *K_sp_ K_f_*, where *K_sp_* is the solubility product constant. For buffer solutions containing equimolal amounts of ammonia and ammonium chloride this quantity amounts to about 0.003, but it is only 0.00011 for the 4-aminopyridine buffers studied here. Inasmuch as the largest correction applied to the ammonia results was about 0.002 in *pw*H [5], it is evident that a negligible error will be incurred by the formation of complexes between silver ion and 4-aminopyridine in the buffer solutions of the present study.

Bruehlman and Verhoek [18] have found that plots of log *K_f_* for amine-silver complexes versus −log *K_bh_* for the base in question consist of a series of straight lines, each characteristic of a different class of base. The data for pyridine and two of its homologues fall on the line of the primary amines rather than that of the tertiary amines. It is of interest to note that our result for 4-aminopyridine also lies close to the line characteristic of the primary amines.

The emf data were corrected in the usual way to a partial pressure of 1 atm of dry hydrogen. The values of the emf summarized in table 1 represent the average of two hydrogen-silver chloride electrode combinations in the same cell. At 25° C, the mean difference between pairs of electrodes in the same cell was 0.06 mv.

## 4. Results

**Figure 1:**
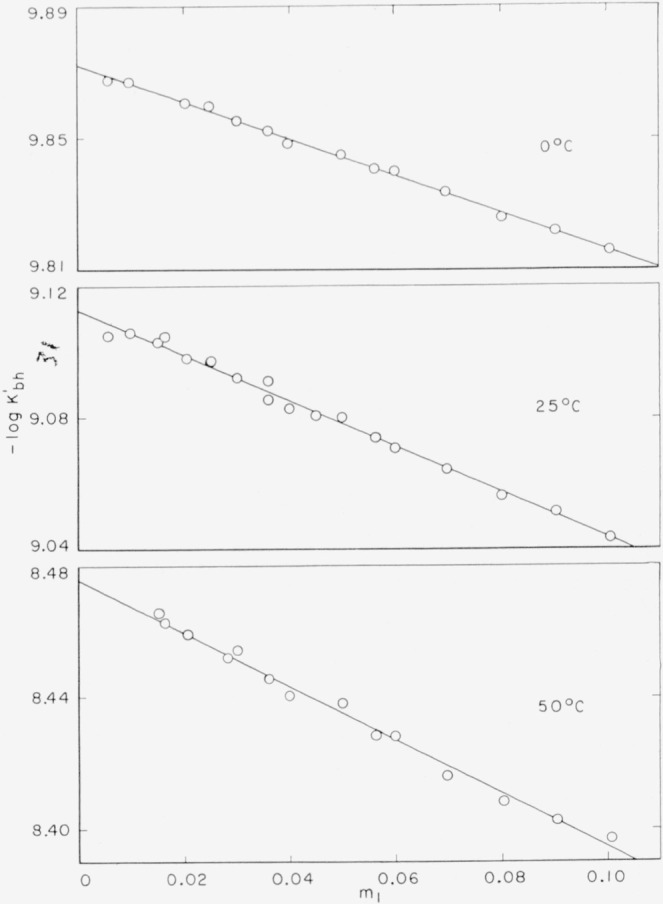
Plots of $-\log\;K_{\mathrm{bh}}^{\prime }$ as a function of *m*_1_ at 0°, 25°, and 50° C.

The values of $-\log\;K_{bh}^{\prime }$ were calculated by eq (5a) and (5b) with several different values of the parameter a*. The best straight-line plots of $-\log\;K_{bh}^{\prime }$ with respect to *m*_1_ at each temperature were obtained with a* = 0. The plots of the data at 0°, 25°, and 50° C are shown in figure 1. The best rectilinear fit of the experimental results was obtained by the method of least squares. The intercepts of the lines at *m*_1_ = 0 gave the values of −log *K_bh_* and *K_bh_*. These are summarized in table 2 along with *σ_i_*, the standard deviation of the intercept, and *K_b_*, the basic dissociation constant of 4-aminopyridine computed by the formula

$$K_{b}=K_{w}/K_{bh}.$$ (7)

It should be noted that *K_b_* is the equilibrium constant for the process

$$B+H_{2}O={\text{BH}}^{+}+{\text{OH}}^{-}.$$ (8)

The values of −log *K_bh_* from the present investigation are compared with earlier determinations in table 3.

## 5. Thermodynamic Quantities

The method of least squares was used to express −log *K_bh_* as a function of absolute temperature by an equation of the form suggested by Harned and Robinson [19], namely

$$-\log\;K_{bh}=\frac{A}{T}+B+CT.$$ (9a)

Between 0° and 50° C, −log *K*_bh_ is given by

$$-\log\;K_{bh}=\frac{2575.8}{T}+0.08277+0.0013093T.$$ (9b)

The constants of this equation were determined by the IBM704 computer. The average difference between “observed” and “calculated values at the 11 temperatures is 0.0014.

By the application of well-known thermodynamic relations, the standard changes of Gibbs free energy (Δ*G*°), enthalpy (Δ*H*°), entropy (Δ*S*°), and heat capacity $\left(\Delta C_{p}^{{}^{\circ}}\right)$ for the dissociation process, eq (1), in the standard state were derived from the constants of eq (9b). The resulting expressions for these quantities are as follows:

$$\Delta G{}^{\circ}=49,313+1.585\;T+0.02507\;T^{2}\;(j\;{\text{mole}}^{-1})$$ (10)

$$\Delta H{}^{\circ}=49,313-0.02507\;T^{2}\;(j\;{\text{mole}}^{-1})$$ (11)

$$\Delta S{}^{\circ}=-1.58-0.0501\;T\;(j\;\deg^{-1}\;{\text{mole}}^{-1})$$ (12)

$$\Delta C_{p}^{{}^{\circ}}=-0.050\;T\;(j\;\deg^{-1}\;{\text{mole}}^{-1}).$$ (13)

The values obtained by these equations are summarized in table 4. The estimated uncertainties at 25°C are as follows: Δ*G*°,±6 j mole ^−1^; Δ*H°,±* 100 j mole ^−1^; Δ*S*°, ±0.5 j deg ^−1^ mole ^−1^; and $\Delta C_{p}^{{}^{\circ}}$, 5 j deg^−1^ mole^−1^.

Elliott and Mason [20] have calculated the heat and entropy of ionization of 4-aminopyridinium ion from values of −log *K_bh_* at three temperatures, namely 5.4°, 20°, and 35° C. Their results are as follows:

$$\Delta H{}^{\circ}\left(20{}^{\circ}\;C\right)=45,000\;j\;{\text{mole}}^{-1};\;\Delta S{}^{\circ}\left(20{}^{\circ}\;C\right)=-24\;j\;\deg^{-1}\;{\text{mole}}^{-1}.$$

The equation for the variation of −log *K_b_* with absolute temperature (*T*) is obtained, as suggested by eq (7), by subtracting the equation for −log *K_bh_* (eq (9b)) from the corresponding equation for −log *K_w_* given by Robinson and Stokes [11]. In this way, one obtains

$$-\log\;K_{b}=\frac{1895.53}{T}-6.1674+0.015744T.$$ (14)

From the constants of this equation, thermodynamic quantities for the basic dissociation of 4-aminopyridine (B) at 25° C were calculated, with the following results:

$$\begin{array}{l} \text{Process}:B+H_{2}O={\text{BH}}^{+}+{\text{OH}}^{-}\;\text{at}\;25{}^{\circ}C\\ \;\Delta G{}^{\circ}=27,880\;j\;{\text{mole}}^{-1}\\ \;\Delta H{}^{\circ}=9,490\;j\;{\text{mole}}^{-1}\\ \;\Delta S{}^{\circ}=-61.7\;j\;{\text{deg}}^{-1}\;{\text{mole}}^{-1}\\ \;\Delta C_{p}^{{}^{\circ}}=-180\;j\;{\text{deg}}^{-1}\;{\text{mole}}^{-1}. \end{array}$$

## 6. Discussion

Recent studies [20 to 22] indicate clearly that it is the nuclear, or pyridine, nitrogen of 4-aminopyridine which has much the greater attraction for the proton. Indeed, Hirayama and Kubota [21] found that the proton does not add in significant amounts to the amino nitrogen even in 50-percent sulfuric acid. They estimated −log *K_bh_* = −6.55 for the dissociation of the bivalent cation acid, ^+^H_3_NC_5_H_4_NH^+^, in 95-percent sulfuric acid.

Pyridine itself is a very weak base (−log *K_bh_* about 5.2) and aniline is still weaker (−log *K_bh_*=4.6). It is of interest to inquire into the reasons for the ten-thousandfold increase in basicity of the nuclear nitrogen atom when an amino group is substituted on the carbon atom located in position 4. Albert, Goldacre, and Phillips [3], in their study of the strength of heterocyclic bases, attribute the increase to increased resonance in the structure of the ion. They point out that analogous compounds in the quinoline and acridine series also show a similar increase.

It is well understood that an uncharged base tends to add a proton if resonant structures imparting greater stability to the cation than to the free base itself exist [23]. The low basicity of aniline can be explained by the fact that the resonance energy of the neutral base is greater than that of the anilinium ion. The resonance structure which makes the strongest contribution appears to be the following:

**Figure:**
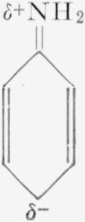

In this resonance configuration, a flow of electrons toward the ring gives rise to a residual positive charge (*δ*^+^) on the amino nitrogen, which in turn repels the proton. No similar resonance contribution is possible in anilinium ion.

On the other hand, the weakly basic nature of pyridine cannot be explained on the basis of resonance. Similar resonance structures can be written for both pyridine and pyridinium ion, so that resonance does not favor the molecular form, as is the case with aniline.

The weakness of pyridine as a base is presumably due to the fact that the nitrogen is doubly bonded to one of the adjacent carbon atoms in each of the Kekulé structures. It can be shown on theoretical grounds that an atom joined to another by a multiple bond holds its remaining electrons more firmly than usual [23]. The donor properties of the nitrogen atom are correspondingly lessened.

The greatly enhanced basicity of the nuclear nitrogen in 4-aminopyridine appears to be the result of a strong contribution of the resonance configuration to the structure of the free base [23, 24].

**Figure:**
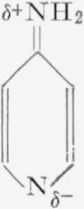

The increased density of charge on the ring nitrogen attracts protons, whereas the residual positive charge of the amino nitrogen repels them so effectively that almost no basicity in the usual sense remains. Resonance structures likewise stabilize the cation effectively.

A comparison of the dipole moments of aniline, pyridine, and 4-aminopyridine offers some support for the existence of the resonant structure in the free base [24]. A simple linear combination of the moments of aniline (1.90 Debye units) and pyridine (2.22 Debye units) in dioxane solution would lead to a moment of 4.12 if the vectors are all in the plane of the ring. The observed moment for 4-aminopyridine in dioxane is 4.36 Debye units, pointing to an appreciable contribution of the resonant configuration. Sidgwick [25] has suggested that 4-aminopyridine can exist in a tautomeric inline form, HN=C_5_H_4_NH.^3^ The symmetry of this tautomer would, however, probably cause the molecule to exhibit only a small dipole moment; hence, the relatively large observed moment would appear to rule out the existence of this tautomer in significant amounts in dioxane solutions of 4-aminopyridine.

The interpretation of the changes of heat content, entropy, and heat capacity that accompany the dissociation of univalent cation acids should be somewhat simpler, in view of the equality of charge distribution among reactants and products, than the interpretation of the corresponding data for uncharged acids or anion acids.^4^ It is nonetheless still exceedingly complex and, furthermore, is hampered by a lack of data for a group of several related, sterically similar, acids.

In terms of the Harned-Robinson equation, eq (9a), the difference between Δ*S*° and $\Delta C_{p}^{{}^{\circ}}$ for a given acid or base is constant and independent of temperature:

$$\Delta S{}^{\circ}-\Delta C_{p}^{{}^{\circ}}=-2.3026RB=-19.1447B.$$ (15)

The value of *B* is positive for the dissociation of most cationic acids (see appendix 12.1 of [11]), and hence $\Delta C_{p}^{{}^{\circ}}$ usually exceeds Δ*S*° for acids of this charge type. The *B* constant for the dissociation of 4- aminopyridinium ion is very small; hence, Δ*S*° and $\Delta C_{p}^{{}^{\circ}}$ are nearly equal. Nevertheless, these two quantities need not always have the same sign; for the dissociation of piperidinium ion [7], Δ*S*° at 25° C was found to be −34 j deg^−1^ mole^−1^ whereas $\Delta C_{p}^{{}^{\circ}}$ was 88 j deg^−1^ mole^−1^ at the same temperature. This decrease of entropy on dissociation, larger than has been found for other acids of this charge type, was attributed to steric exclusion of solvent from the immediate vicinity of the piperidinium ion. Consequently, the entropy-increasing effect of the release of water molecules on dissociation is absent. If it be assumed that the two free bases are equally solvated, it may be reasoned that the wider distribution of charge possible with the existence of resonant forms permits more extensive solvation of 4-aminopyridinium ion than occurs with piperidinium ion.

If changes in solvent orientation play an important part in determining the sign and magnitude of the entropy change on dissociation, the same should be true of the heat capacity change: release of water from combination would be expected to bring about an increase in both entropy and heat capacity. Structural changes, however, sometimes result in an increase in one of these quantities and a decrease in the other. Unfortunately, no independent assessment of the degree of solvation of the acidic and basic forms is available, and our knowledge of the details of the geometry of the molecules and ions and their immediate surroundings is likewise incomplete. Thus, measurements of the entropies and heat capacities for dissociation reactions are as yet unable alone to provide an insight into the mechanisms of acid-base behavior.

## Figures and Tables

**Table 1:** Electromotive force of the cell: *Pt*; *H*_2_(g, 1 *atm*), *H*_2_*NC*_5_*H*_4_*N*·*HCl* (m_1_), *H*_2_*NC*_5_*H*_4_*N* (m_2_), *AgCl*; *Ag* from 0° to 50° C, in volts

*m*_1_	*m*_2_	0°	5°	10°	15°	20°	25°	30°	35°	40°	45°	50°
												
0.10066	0.09914	0.83898	0.83849	0.83784	0.83704	0.83611	0.83499	0.83384	0.83255	0.83113	0.82960	0.82801
.09031	.08919	.84105	.84058	.83987	.83913	.83827	.83732	.83615	.83485	.83341	.83193	.83032
.08005	.07884	.84311	.84270	.84213	.84143	.84058	.83958	.83829	.83707	.83571	.83425	.83279
.06961	.06875	.84588	.84547	.84492	.84421	.84341	.84256	.84165	.84037	.83885	.83747	.83594
.05993	.05902	.84868	.84827	.84784	.84715	.84651	.84559	.84460	.84348	.84227	.84097	.83952
.05625	.05310	.84880	.84845	.84801	.84741	.84659	.84585	.84482	.84368	.84247	.84113	.83960
.04985	.04923	.85221	.85187	.85149	.85094	.85038	.84964	.84869	.84766	.84653	.84528	.84390
.04505	.04554	……….	……….	……….	……….	……….	.85222	.85123	……….	……….	……….	……….
.03986	.03926	.85635	.85618	.85589	.85542	.85482	.85406	.85325	.85229	.85109	.84987	.84858
.03604	.03402	.85741	.85722	.85689	.85645	.85584	.85512	.85436	……….	……….	……….	……….
.03597	.03637	……….	……….	……….	……….	……….	.85726	.85627	.85538	.85436	.85337	.85184
.02996	.02959	.86209	.86199	.86183	.86145	.86097	.86041	.85972	.85888	.85785	.85652	.85569
.02492	.02352	.86482	.86466	.86453	.86431	.86388	.86339	.86251	.86175	.86071	.85963	.85839
.02466	.02493	……….	……….	……….	……….	……….	.86534	.86437	.86358	.86268	.86181	……….
.02003	.019730	.87014	.87006	.86996	.86986	.86964	.86914	.86872	.86793	.86699	.86583	.86498
.016111	.016289	……….	……….	……….	……….	……….	.87492	.87435	.87360	.87291	.87238	.87102
.014963	.014126	……….	……….	……….	……….	……….	.87468	.87403	.87334	.87269	.87189	.87104
.009766	.009646	.88520	.88529	.88562	.88576	.88575	.88556	.88561	.88504	.88437	……….	……….
.005725	.005405	.89551	.89582	.89630	.89655	.89669	.89667	……….	……….	……….	……….	……….

**Table 2:** Summary of the dissociation constants of 4-amino- pyridinium ion and 4-aminopyridine from 0° to 50° C

Temperature	−log *K_bh_*	*σ_i_*	*K_bh_×*10^10^	−log *K_b_*	*K_b_*×10^5^
					
*°C*					
0	9.8731	0.0005	1.339	5.070	0.851
5	9.7043	.0006	1.976	5.030	.934
10	9.5486	.0006	2.827	4.986	1.03
15	9.3979	.0006	4.000	4.948	1.13
20	9.2524	.0006	5.592	4.915	1.22
25	9.1141	.0007	7.690	4.882	1.31
30	8.9783	.0012	10.51	4.855	1.40
35	8.8455	.0010	14.27	4.834	1.47
40	8.7170	.0013	19.19	4.818	1.52
45	8.5941	.0014	25.46	4.802	1.58
50	8.4768	.0014	33.36	4.785	1.64

**Table 3:** Comparison of −log *K_bh_* with earlier determinations at 4 temperatures

Authors; method	5.4° C	20° C	25° C	35° C
				
Tropsch [2]; conductance	……….	……….	9.11	……….
Van Hall and Stone [1]; potentiometric titration	……….	……….	9.11	……….
Hansson [3]; conductance	……….	9.24	……….	……….
Albert, Goldacre, and Phillips [4]; potentiometric titration	……….	a9.29	……….	……….
Elliott and Mason [20]; potentiometric titration	9.74	……….	……….	8.93
This investigation; emf	9.692	9.252	9.114	8.845

a Corrections for dilution applied in the manner prescribed by the authors.

**Table 4:** Thermodynamic quantities for the acidic dissociation of 4-aminopyridinium ion from 0° to 50° C

*t*	Δ*G*°	Δ*H*°	Δ*S*°	$\Delta C_{p}^{{}^{\circ}}$
				
*°C*	*j mole*^−1^	*j mole*^−1^	*j deg*^−1^ *mole*^−1^	*j deg*^−1^ *mole*^−1^
0	51,616	47,440	−15.3	−14
5	51,693	47,370	−15.5	−14
10	51,771	47,300	−15.8	−14
15	51,851	47,230	−16.0	−14
20	51,931	47,160	−16.3	−15
25	52,013	47,090	−16.5	−15
30	52,097	47,010	−16.8	−15
35	52,181	46,930	−17.0	−15
40	52,267	46,860	−17.3	−16
45	52,354	46,780	−17.5	−16
50	52,442	46,700	−17.8	−16

## Footnotes

1 Figures in brackets indicate the literature references at the end of this paper.

2 The authors are indebted to M. M. Davis and M. Paabo for the initia purification.

3 The existence of the imine structure has also been suggested by Mason [26].

4 As the Born equation shows, however, the electrostatic work of charging two spherical ions of the same charge is only equal when the radii of the ions are the same.
